# Supplementary material for: An intermittent detachment faulting system with a large sulfide deposit revealed by multi-scale magnetic surveys
Source: Nat Commun. 2021 Sep 24;12:5642. doi: 10.1038/s41467-021-25880-1 (PMC8463574; doi:10.1038/s41467-021-25880-1)
Supplement: Supplementary file 1 — Supplementary Information [file 41467_2021_25880_MOESM1_ESM.pdf]

An intermittent detachment faulting system with a large sulfide deposit revealed by multi-scale magnetic surveys

Tao Wu et al.

List of Supplementary materials

Supplementary Figure 1. Detailed structures showed by high-resolution bathymetry and geology information including lithology of samples and hydrothermal vents distribution in detachment fault area.
Supplementary Figure 2. Near-bottom magnetic anomalies and results of magnetization inversion in the LQ-1 hydrothermal vent area.
Supplementary Figure 3. High resolution bathymetry by ABE201 and the distribution of hydrothermal vents of the LQ-1 vent site as mapped by the HOV *Jiaolong*.
Supplementary Table 1. The lithologic characters and magnetization of samples in Dragon Horn collected by TV grab.
Supplementary Note 1. Magnetic features of the hydrothermal areas.

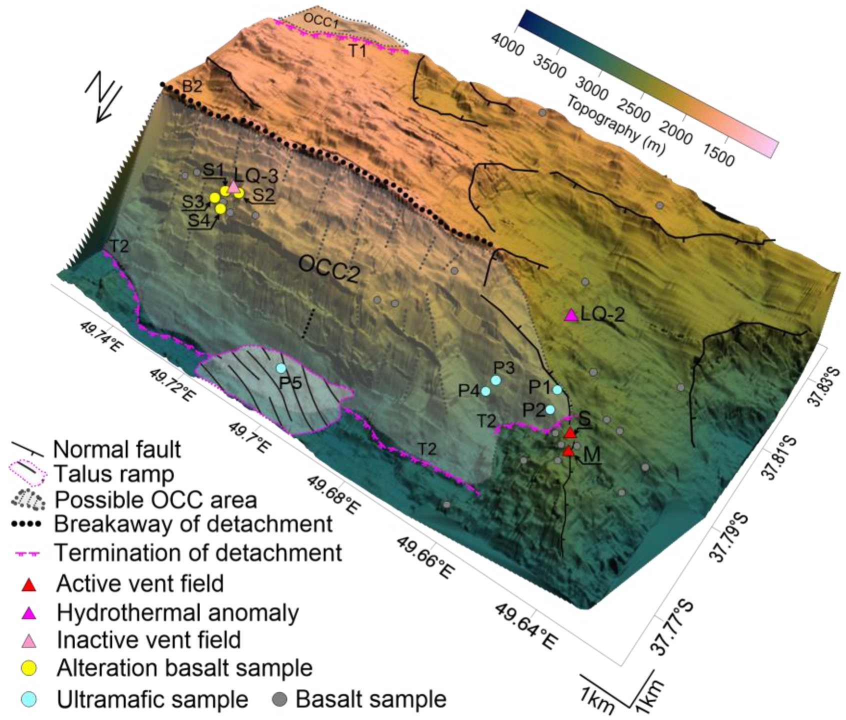

Supplementary Figure 1. Detailed structures showed by high-resolution bathymetry and geology information including lithology of samples and hydrothermal vents distribution in detachment fault area.

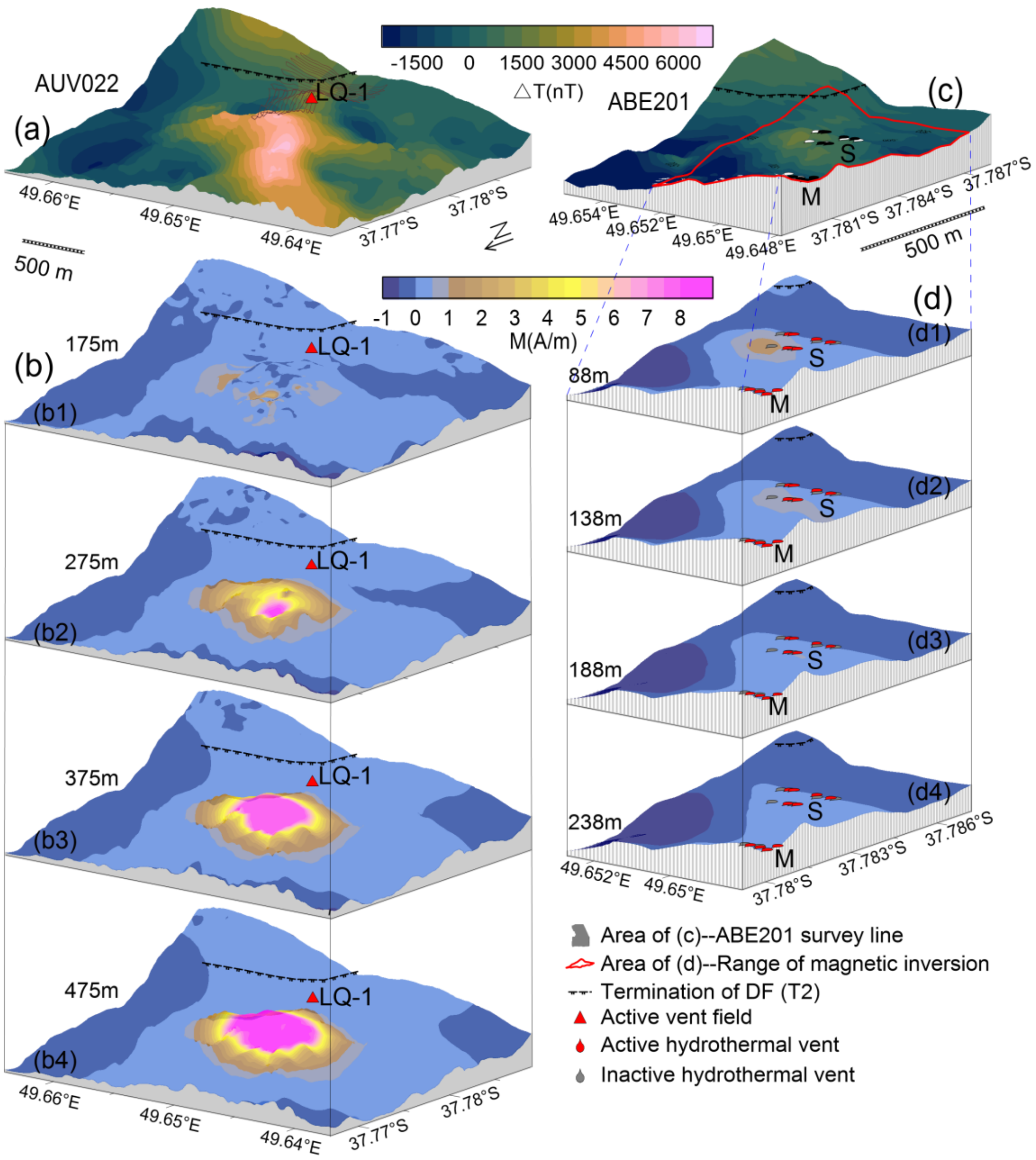

Supplementary Figure 2. Near-bottom magnetic anomalies (a) and (c), and results of magnetization inversion (b) and (d) in the LQ-1 hydrothermal vent area. (a) Magnetic anomalies of AUV022 and (b) are the corresponding 3-D magnetization structure in depth with 175 m (b1), 275 m (b2), 375 m (b3) and 475 m (b4), respectively. (c) Magnetic anomalies of ABE201 and (d) are 3-D magnetization inversion results of vents zone M and S (circled by black box in (c)) in depth with 88 m (d1), 138 m (d2), 188 m (d3) and 238 m (d4), respectively. Magnetization direction is assumed to be parallel to the geocentric axial dipole (inclination and declination of -62.3° and -42.0°, respectively).

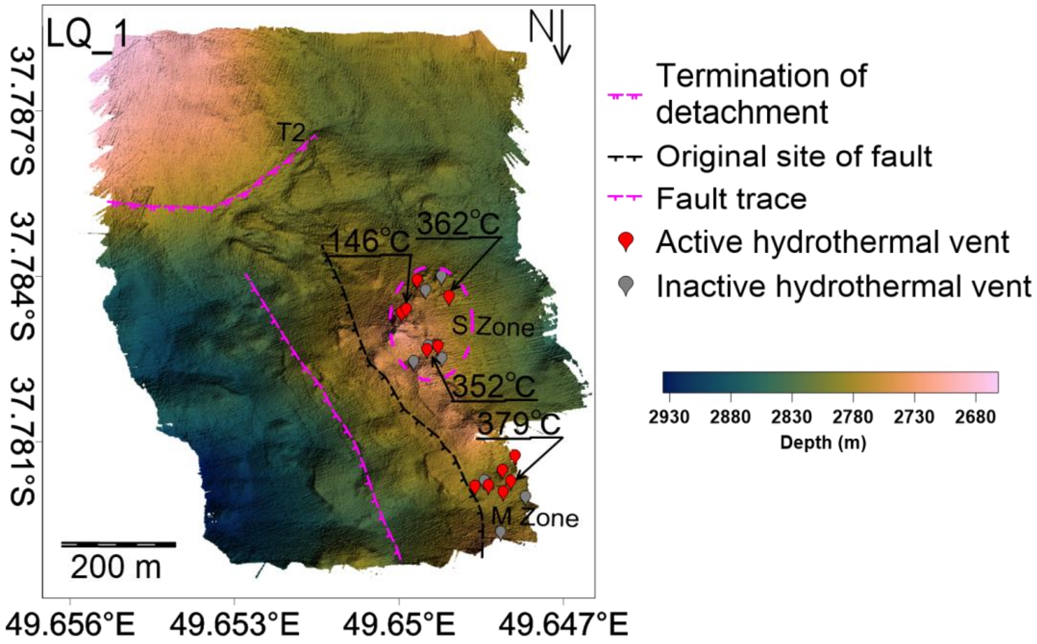

Supplementary Figure 3. High resolution bathymetry by ABE201 and the distribution of hydrothermal vents of the LQ-1 vent site as mapped by the HOV *Jiaolong*. Active vent field temperatures are noted.

Supplementary Table 1. The lithologic characters and magnetization of samples in Dragon

Area	Station No.	Rock types (bolder words) and microscopic features of parts samples	Magnetization （A m^{-1}）
LQ-1	P1	**Serpentinite (20%)** composes by residual granules of olivine and reticulated veins of serpentine. The network structure can be seen in section.	1.95, 2.03, 1.35, 2.2 (4 samples of Serpentinite)
		**Altered spherolitic basalt (80%)** has visible structure of microcracks and pores. Chlorite and a small amount of quartz filled in cracks.	
	P2	**Serpentinizated peridotite (40%)** with network structure, composes by serpentine and residual peridot after alteration.	——
		**Basalt (60%)** suffered low degree of fragmentation. Chlorite or quartz can be seen in cracks of basalt under the microscope.	
	P3	**Altered pyroxene** presents greyish-green to light green at its surface and has wide-spread of fibrous talc particles.	——
		**Serpentine** largely replaced pyroxene and the crystal outline of original mineral is not visible**.** The aggregate of serpentine presents fold shape.	
	P4	**Gabbroic and altered pyrogenic protomylonite** has mylonitic texture. Broken plagioclase and oriented pyroxene is visible.	——
		**Subamphibolite pyroxenite**, with cataclastic and residual columnar texture, suffered strongly altered and formed ouralite and serpentine. Chlorite and epidote were filled in crack.	
		**Serpentine** with network structure mainly composed by chrysotile.	
Talus ramp	P5	**Basalt** has intergranular texture and its phenocryst mainly composes by olivine and plagioclase.	——
		**Altered cataclastic gabbro** has cataclastic and gabbro texture.	
		**Altered pyroxenite** had a high degree of fragmentation and accompanied with amphibolization. Chlorite and epidote were filled in crack.	
		**Serpentine** has network structure. Magnetite and other opaque iron mineral distributed.	
LQ-3	S1	**Altered basalt** has brecciated texture and contains cementation of sulfide with metallic luster. Microscopically, aggregation of radial subamphibole is visible as basalt was altered. Meanwhile, the rock formed larger cracks where quartz and opaque iron mineral with needle-columnar or granular was filled in under the influence of structure stress	40.60, 19.90
	S2	**Variolite** with intersertal texture composed of acicular plagioclase with puce and buff basalt glass filled in gap and the chlorite veins is visible.	——
	S3	**Altered basalt** was ruptured after effected by structure stress and then formed ring-like structure under the influence of hydrothermal alteration	1.90, 2.56
	S4	**Cataclastic altered basalt** was greatly ruptured and amount of chlorite was filled in basaltic detritus.	4.83, 2.61, 3.62
	S5	**Diabase** mainly contains plagioclase and pyroxene. Chlorite and opaque iron mineral was filled in framework.	2.63, 2.72, 2.40 (Basalt)
		**Vesicular altered basalt** has variolite texture and contains microcrystalline of radial feldspar. Chlorite was filled in vesicle.	
*Notes	‘——’ represents no work on magnetization measurement		

Supplementary Note 1. Magnetic features of the hydrothermal areas

LQ-2 is a hydrothermal plume anomaly and very little is known yet about this site and its exact location. Similarly, the Longjing hydrothermal sites (LJ-E, LJ-W) are located near the older OCC-1 or on strike with the OCC at the edge of the ridge segment (Fig. 1b) and little is known about them at this time. Thus, we focus on the better defined LQ-3 and LQ-1 hydrothermal vent fields and discuss below their detailed magnetic settings and how they relate to the OCC structure.

LQ-3 vent field - The inactive vent field LQ-3 appears to have been originally part of the footwall but is now a slipped hanging wall block that is marked by basaltic basement. Rock samples reveal that a significant amount of breccia and/or altered basalt are distributed in the area surrounding the LQ-3 vent, suggesting that hydrothermal fluid has altered the host rock when the vent was active in the past. While we would expect to see a zone of reduced magnetization associated with this hydrothermal area[1], we do not observe an obvious magnetic low on the magnetic map (Fig. 3a). We speculate that this is because the AUV survey line spacing was too wide (400 m) and the grid interpolation is not sensitive enough to detect features on the scale of ~100 m. However, by extracting the raw profile data (Fig. 3b) that crosses the LQ-3 vent site from east to west (marked by double arrow line in Fig. 3a) we find a narrow but obvious magnetic anomaly low over the LQ-3 vent site with about 200 m width (circled by red dotted line in Fig. 3b). Given the recovery of altered basalt from the site, we conclude that the focused hydrothermal alteration has influenced the basaltic crust locally in the vicinity of the LQ-3 vent site.

LQ-1 vent field - The regional AUV022 survey (see Fig. 3a, Supplementary Figure 2a) shows that a strong magnetic anomaly is present over the hanging wall block below the termination. The active LQ-1 vent field sits just at the southern edge of this strong magnetic zone on the hanging wall block but just north of the termination. The adjacent OCC-2 fault shows magnetic anomaly lows above the termination that are a continuation of magnetic lows that mark the main OCC-2 exposure. A magnetic high associated with the breakaway B2 wraps around the northern edge of the OCC and stops at the termination just before the LQ-1 vent field (Fig. 3a, Supplementary Figure 2a). The focused 3D magnetization inversion of the LQ-1 vent field area (Supplementary Figure 2b) shows that the zone of highest magnetization forms a bulls-eye positive anomaly just north of the LQ-1 vent field (Supplementary Figure 2b). We infer that this strong magnetization is the result of intrusive emplacement of magma into this block from a nearby heat source. We note that the non-transform discontinuity is nearby and projects into this location suggesting a possible link to recent magmatism (Fig. 1a). While the 400 m line spacing of the AUV022 survey is too wide to capture the detailed magnetic response of the LQ-1 vent field directly, the vent field was surveyed previously by

the AUV ABE[2] with a line spacing of 30 m (Supplementary Figure 2c). The ABE dive 201 data were reprocessed and inverted with the focused 3D inversion method (Supplementary Figure 2d) but it does not show any clear magnetic low associated with the vent field and simply shows a relatively weak magnetization throughout the area that does not vary much with depth – at least to 238 m. One possible interpretation of this result suggests that there has been pervasive alteration in this region such that no large magnetization contrasts are present in the upper part of the crust.

Supplementary references

1. Tivey, M. A., & Dyment, J. The magnetic signature of hydrothermal systems in slow spreading environments. Diversity of Hydrothermal Systems on Slow Spreading Ocean Ridges, *Geophys. Monogr. Ser*, **188**, 43-65 (2010).
2. Zhu, J. et al. A reduced crustal magnetization zone near the first observed active hydrothermal vent field on the Southwest Indian Ridge. *Geophys. Res. Lett.* **37**, n/a-n/a (2010).
